# Supplementary material for: Influenza Vaccination in Italian Healthcare Workers (2018–2019 Season): Strengths and Weaknesses. Results of a Cohort Study in Two Large Italian Hospitals
Source: Vaccines (Basel). 2020 Mar 5;8(1):119. doi: 10.3390/vaccines8010119 (PMC7157508; doi:10.3390/vaccines8010119)
Supplement: Supplementary file 1 [file vaccines-08-00119-s001.pdf]

Table S1: Questionnaire for data collection of demographic characteristics, chronic conditions, risk factors and vaccination status.

Code Region
Code Hospital
Code Subject

SECTION A: Data of the subject

Date of birth: |__|__|____|
Surname: |__________________________| Name: |__________________________|
Sex: F |__| M |__|

SECTION B: Vaccination

Who provides information on the subject's vaccination status?

ASL or Hospital |__| GP |__|

Did the patient receive vaccination against seasonal flu in 2018-2019? Yes |__| No |__|
If yes, write the vaccination date: |__|__|____|
If yes, indicate the commercial name of the vaccine: |____|
Did the patient receive vaccination against seasonal flu in 2017-2018? Yes |__| No |__| Don’t know |__|
Did the patient receive vaccination against seasonal flu in 2016-2017? Yes |__| No |__| Don’t know |__|
Did the patient receive the 23-valent pneumococcal vaccine? Yes |__| No |__| Don’t know |__|
Did the patient receive 13-valent conjugate pneumococcal vaccine? Yes|__| No |__| Don’t know |__|

SECTION C: chronic diseases

Chronic pulmonary disease [1] : Yes |__|No |__| ND|__|
Cardiovascular disease [2]: Yes |__|No |__| ND|__|
Diabetes mellitus and other metabolic diseases: Yes |__|No |__| ND|__|
Kidney diseases with renal failure: Yes |__|No |__| ND|__|
Diseases of hematopoietic organs and hemoglobinopathies: Yes |__|No |__| ND|__|
Cancer: Yes |__|No |__| ND|__|
Severe liver disease and cirrhosis of the liver: Yes |__|No |__| ND|__|
Congenital and acquired diseases that impair antibody production: Yes |__|No |__| ND|__|
Immunosuppression induced by Drugs or HIV: Yes |__|No |__| ND|__|
Chronic inflammatory diseases
and intestinal malabsorption syndromes: Yes |__|No |__| ND|__|

Pathologies associated with an increased risk of
aspiration of respiratory secretions [3]: Yes |__|No |__| ND|__|
Obesity with Body mass index (BMI) > 30
and severe medical comorbidities: Yes |__|No |__| ND|__|
Anemia, enlargement of the spleen: Yes |__|No |__| ND|__|
Leukemia, lymphomas: Yes |__|No |__| ND|__|

[1] including asthma, bronchopulmonary dysplasia, cystic fibrosis and COPD.
[2] including congenital and acquired heart disease.

[3]for example neuromuscular diseases.

Nutritional deficiencies: Yes |__|No |__|ND|__|
Dementia / stroke: Yes |__|No |__| ND|__|
Rheumatic diseases: Yes |__|No |__| ND|__|

ND= not defined

SECTION D: Risk factors

Does the patient have hypertension or hypercholesterolemia in therapy? Yes |__| No |__| Don't know |__|
How many times has the patient been hospitalized for the diseases indicated in Section C in the previous 12 months?[4] |____| *(0 if none)*
Does the patient smoke? Never |__| Ex[5] |__| Yes |__| Don't know |__|
Is the patient pregnant? Yes|__| No |__| Don't know |__|

[4] described in Section C "Chronic diseases".

[5] The patient has stopped smoking for at least one year

Table S2. Questionnaire for clinical data.

In the case of Influenza Like Illness (ILI)**

Date of symptoms onset: |__|__|____| *(if started more than seven days NOT to be reported)*

SECTION E: Symptoms

Sudden onset of symptoms: Yes |__| No |__|
Fever or feverishness: Yes |__| No |__|
Malaise: Yes |__| No |__|
Headache: Yes |__| No |__|
Myalgia: Yes |__| No |__|
Cough: Yes |__| No |__|
Sore throat: Yes |__| No |__|
Shortness of breath: Yes |__| No |__|
Other ________

Naso or oro-pharyngeal swab

Date of swabbing: |__|__|____|

Unique identification code generated by the Web system

SECTION F: Antiviral therapy (data not mandatory)

Has the patient taken antiviral drugs? Yes* |__| No |__|
If yes, indicate the date of administration of the drug: |__|__|____|
If yes, indicate the commercial name: |____|
Oseltamivir-Tamiflu/Zanamivir-Relenza

* non-mandatory information

** A case of ILI is defined, in accordance with the ECDC case definition, as an individual who presents with a sudden onset of symptoms. Including at least one of the following systemic symptoms: fever or feverishness; malaise; headache; myalgia. And at least one of the following three respiratory symptoms: cough; sore throat; and shortness of breath.

Table S3. Characteristics and vaccination status of enrolled subjects in Genoa hospital.

Age group (years)	18-24 (%)	25-34 (%)	35-44 (%)	45-54 (%)	55-64 (%)	≥65 (%)	Total (%)
**Gender**							
F	303 (18.7)	331 (20.5)	193 (11.9)	450 (27.8)	290 (17.9)	50 (3.1)	1617 (100)
M	110 (16.8)	188 (28.8)	74 (11.3)	122 (18.7)	131 (20.1)	28 (4.3)	653 (100)
Total	413 (18.2)	519 (22.9)	267 (11.8)	572 (25.2)	421 (18.5)	78 (3.4)	2270 (100)
**Any chronic condition**							
0	358 (86.7)	458 (88.2)	236 (88.4)	435 (76.0)	292 (69.4)	54 (69.2)	1833 (80.7)
≥1	55 (13.3)	61 (11.8)	31 (11.6)	137 (24.0)	129 (30.6)	24 (30.8)	437 (19.3)
**Risk factor**							
Smoking	96 (23.2)	118 (22.7)	50 (18.7)	139 (24.3)	105 (24.9)	8 (10.3)	516 (22.7)
**Number of hospitalizations in the previous 12 months**							
0	407 (98.5)	511 (98.5)	264 (98.9)	560 (97.9)	410 (97.4)	74 (94.9)	2226 (98.1)
≥1	6 (1.5)	8 (1.5)	3 (1.1)	12 (2.1)	11 (2.6)	4 (5.1)	44 (1.9)
**Influenza vaccination status in previous season (2017-2018)**							
Vaccinated	24 (5.8)	80 (15.4)	27 (10.1)	51 (8.9)	70 (16.6)	26 (33.3)	278 (12.4)
Unvaccinated	328 (79.4)	357 (68.8)	193 (72.3)	411 (71.9)	252 (59.9)	38 (48.7)	1579 (69.6)
Unknown	61 (14.8)	82 (15.8)	47 (17.6)	110 (19.2)	99 (23.5)	14 (17.9)	413 (18.2)
**Influenza vaccination status in season 2018-2019**							

Vaccinated	123 (29.8)	238 (45.9)	84 (32.5)	171 (29.9)	174 (43.3)	40 (51.3)	830 (36.6)
Unvaccinated	290 (70.2)	281 (54.1)	183 (68.5)	401 (70.1)	247 (63.4)	38 (48.7)	1440 (63.5)
Total	413 (100)	519 (100)	267 (100)	572 (100)	421 (100)	78 (100)	2270 (100)
**Vaccine brands among the vaccinated in season 2018-2019**							
Agrippal®	1 (0.8)	-	-	-	1 (0.6)	-	2 (0.2)
Fluad®	-	-	-	-	3 (1.7)	20 (50.0)	23 (2.8)
Fluarix Tetra®	117 (95.1)	218 (91.6)	79 (94.0)	155 (91.2)	157 (89.7)	18 (45.0)	744 (89.6)
Vaxigrip Tetra®	5 (4.1)	20 (8.4)	5 (6.0)	15 (8.8)	14 (8.0)	-	59 (7.1)
Unknown	-	-	-	-	-	2 (5.0)	2 (0.2)

Table S4. Characteristics and vaccination status of enrolled subjects in Milan hospital

**Age group (years)**	**18-24 (%)**	**25-34 (%)**	**35-44 (%)**	**45-54 (%)**	**55-64 (%)**	**≥65 (%)**	**Total (%)**
**Gender**							
F	360 (23.3)	416 (26.9)	219 (14.2)	317 (20.5)	201 (13.0)	31 (2.0)	1544 (100)
M	140 (20.9)	226 (33.8)	92 (13.8)	89 (13.3)	102 (15.2)	20 (3.0)	669 (100)
Total	500 (22.6)	642 (29.0)	311 (14.1)	406 (18.3)	303 (13.7)	51 (2.3)	2213 (100)
**Any chronic condition**							
0	469 (93.8)	588 (91.6)	274 (88.1)	331 (81.5)	233 (76.9)	36 (70.6)	1931 (87.3)
≥1	31 (6.2)	54 (8.4)	37 (11.9)	75 (18.5)	70 (23.1)	15 (29.4)	282 (12.7)
**Risk factor**							
Smoking	90 (18.0)	136 (21.2)	72 (23.2)	74 (18.2)	73 (24.1)	7 (13.7)	452 (20.4)
**Number of hospitalizations in the previous 12 months**							
0	498 (99.6)	639 (99.5)	311 (100)	397 (97.8)	292 (96.4)	48 (94.1)	2185 (98.7)
≥1	2 (0.4)	3 (0.5)	-	9 (2.2)	11 (3.6)	3 (5.9)	28 (1.3)
**Influenza vaccination status in previous season (2017-2018)**							
Vaccinated	43 (8.6)	104 (16.2)	62 (19.9)	69 (17.0)	62 (20.5)	26 (51.0)	366 (16.5
Unvaccinated	451 (90.2)	532 (82.9)	240 (77.2)	331 (81.5)	231 (76.2)	24 (47.1)	1809 (81.7)
Unknown	6 (1.2)	6 (0.9)	9 (2.9)	6 (1.5)	10 (3.3)	1 (2.0)	38 (1.7)
**Influenza vaccination status in current season 2018-2019**							
Vaccinated	113 (22.6)	191 (29.8)	91 (29.3)	102 (25.1)	103 (34.0)	29 (56.9)	629 (28.4)

Unvaccinated	387 (77.4)	451 (70.2)	220 (70.7)	304 (74.9)	200 (66.0)	22 (43.1)	1584 (71.6)
**Vaccine brands among the vaccinated in season 2018-2019**							
Agrippal®	4 (3.5)	-	1 (1.1)	-	1 (1.0)	-	6 (1.0)
Fluad®	-	-	-	1 (1.0)	2 (1.9)	1 (3.4)	4 (3.8)
Fluarix Tetra®	5 (4.4)	10 (5.2)	2 (2.2)	2 (2.0)	4 (3.9)	1 (3.4)	24 (3.8)
Vaxigrip Tetra®	104 (92.0)	181 (94.8)	88 96.7)	99 (97.1)	96 (93.2)	27 (93.1)	595 (94.6)
Unknown	-	-	-	-	-	-	-

Table S5. Association of demographic and clinical characteristics in subjects with influenza

	**Unadjusted HR**	**Adjusted HR**
	**(95% CI); p**	**(95% CI); p**
**Influenza**		
**Gender** (Male vs Female)	0.81 (0.48-1.38); p=0.43	0.78 (0.46-1.32); p=0.35
**Age (years)**		
18-24	1.00 (ref)	1.00 (ref)
25-34	3.98 (1.53-10.41); p=0.005	3.52 (1.34-9.23); p=0.011
35-44	2.54 (0.83-7.75); p=0.10	2.63 (0.88-7.89); p=0.085
45-54	2.63 (0.95-7.29); p=0.064	2.47 (0.88-6.93); p=0.085
55-64	3.81 (1.39-10.49); p=0.010	3.31 (1.18-9.29); p=0.023
≥65	5.70 (1.53-21.22); p=0.009	3.82 (0.99-14.71); p=0.051
**Any chronic condition** (≥1 vs 0)	1.66 (0.96-2.86); p=0.069	1.65 (0.95-2.87); p=0.077
**Previous influenza vaccination**	2.79 (1.73-4.51); p<0.001	1.54 (0.78-3.05); p=0.21
**N. hospitalization** (≥1 vs 0)	0.86 (0.12-6.16); p=0.88	0.49 (0.07-3.68); p=0.49
**Lombardia vs Liguria**	1.18 (0.74-1.88); p=0.48	1.03 (0.64-1.64); p=0.92
**A(H1N1) pdm09**		
**Gender** (Male vs Female)	0.53 (0.24-1.22); p=0.14	0.52 (0.23-1.19); p=0.12
**Age**		
18-24	1.00 (ref)	1.00 (ref)
25-34	10.4 (1.36-79.3); p=0.024	10.4 (1.35-80.2); p=0.024
35-44	4.76 (0.50-45.78); p=0.18	4.47 (0.47-43.2); p=0.19
45-54	9.40 (1.20-73.4); p=0.033	8.64 (1.10-67.9); p=0.04
55-64	11.5 (1.45-90.4); p=0.021	11.2 (1.40-89.2); p=0.023
≥65	14.2 (1.28-156.2); p=0.03	13 (1.15-147.9); p=0.038
**Any chronic condition** (≥1 vs 0)	2.14 (1.06-4.32); p=0.033	2.10 (1.02-4.32); p=0.044

**Previous influenza vaccination**	1.62 (0.79-3.34); p=0.19	2.05 (0.71-5.90); p=0.19
**N. hospitalization** (≥1 vs 0)	NC	NC
**Lombardia vs Liguria**	1.57 (0.82-3.02); p=0.17	1.47 (0.76-2.85); p=0.26
**A(H3N2) pdm09**		
**Gender** (M vs F)	1.19 (0.58-2.45); p=0.64	1.01 (0.49-2.09); p=0.99
**Age**		
18-24	1.00 (ref)	1.00 (ref)
25-34	2.39 (0.77-7.41); p=0.13	1.79 (0.57-5.63); p=0.32
35-44	1.98 (0.53-7.36); p=0.31	1.78 (0.47-6.79); p=0.40
45-54	0.94 (0.23-3.74); p=0.93	0.92 (0.23-3.78); p=0.91
55-64	1.90 (0.54-6.75); p=0.32	1.49 (0.40-5.54); p=0.55
≥65	3.59 (0.66-19.6); p=0.14	1.75 (0.30-10.1); p=0.53
**Any chronic condition** (≥1 vs 0)	1.17 (0.48-2.84); p=0.72	1.00 (0.40-2.55); p=0.99
**Previous influenza vaccination**	4.85 (2.45-9.59); p<0.001	1.60 (0.66-3.88); p=0.30
**N. hospitalization** (≥1 vs 0)	1.90 (0.26-13.9); p=0.53	1.58 (0.19-12.91); p=0.67
**Lombardia vs Liguria**	0.85 (0.43-1.70); p=0.65	0.66 (0.33-1.32); p=0.24
